# Suboptimal dietary knowledge predicts lower diet quality for cancer prevention among university students in Beirut

**DOI:** 10.1371/journal.pone.0315911

**Published:** 2025-01-03

**Authors:** Jana Jabbour, Rodeina Dandache, Maryam Al Slaybe, Lama Haisam Mattar, Rana Rizk

**Affiliations:** 1 Department of Nutrition and Food Science, School of Arts and Sciences, Lebanese American University, Beirut, Lebanon; 2 Department of Nutrition and Food Science, School of Arts and Sciences, Lebanese American University, Byblos, Lebanon; 3 Institut National de Santé Publique, d’Epidémiologie Clinique, et de Toxicologie (INSPECT-LB), Lebanon; Canadian University Dubai, UNITED ARAB EMIRATES

## Abstract

University students are at a pivotal stage of shaping cancer risk factors. Little is known about their dietary behavior in Lebanon, a country heavily burdened by cancer. This cross-sectional study assessed the dietary knowledge of and adherence to cancer prevention guidelines among university students in Beirut, Lebanon. We hypothesized that students would exhibit low knowledge, poor diet quality, and that knowledge predicted diet quality. Dietary knowledge was explored using a dedicated questionnaire, with scores above the 60^th^ percentile considered as Knowledgeable (Kn+), and those below as less knowledgeable (Kn-). Dietary adherence to cancer prevention guidelines and the predictors of the Alternative Healthy Eating Index (AHEI)- a measure of diet quality calculated using the Modified Mediterranean Prime Screen, were also examined. The sample included 300 participants (55% females, mean age: 20 years). The mean knowledge score was 49.5%. Over 50% of students were aware of the association between red and processed meat, sodium, fruits and vegetables, obesity, and cancer. Kn+ group had a higher intake of vegetables and a lower intake of meats and sweetened beverages. Increased knowledge (B = 0.78, 95%CI: 0.18,1.37) and high physical activity (B = 4.62, 95%CI: 1.66,7.59) were associated with elevated AHEI scores. A significant positive interaction was observed between knowledge and enrollment in a health-related major. University students’ dietary knowledge of and adherence to cancer prevention guidelines are suboptimal. Although higher knowledge predicts high-quality diets, the association was weak. Further studies should investigate the food systems influencing university students’ dietary intake of university students in Lebanon and identify effective interventions to enhance health behavior.

## 1. Introduction

Cancer is a leading determinant of mortality and morbidity worldwide [1, 2]. Lebanon has among the highest prevalence rates in the Middle East region [3]. Recent assessments indicates a 161% rise in cancer incidence in 2020 compared with 2004 [1, 4]. Smoking, alcohol consumption, obesity, sedentary lifestyle, and air pollution are leading contributors to cancer burden in the world, the Middle East and North Africa, including Lebanon [5–7]. The American Cancer Society developed a set of recommendations for cancer prevention, that includes altering eating habits, consuming less alcohol, increasing physical activity, and maintaining a healthy body weight [8, 9].

University students constitute a unique subpopulation of interest for the study of cancer prevention knowledge and behavior [10]. At this pivotal stage of development, they are establishing health habits, which can have long lasting impact on their wellbeing [11]. This population is particularly prone to mental health challenges and to falling preys to substance and food abuse [12]. These factors underscore the importance of fostering cancer risk awareness, which is expected to translate into improved dietary behavior and a reduction in cancer incidence [13]. Yet, the association between knowledge and diet behavior in relevance to cancer prevention is underexplored in Lebanon and the Middle East region, especially among young adults. Studies in Lebanon revealed dietary shifts toward a more Western diet in the general population [14, 15]. Among university students, low adherence to the Mediterranean diet was reported, potentially increasing non communicable disease risks [13, 15, 16]. While adherence to recommendations relevant to olive oil and legumes was high, lower rates were noted for other food groups, especially fruits, nuts, fish, and sweetened beverages. Evidence also pinpoints gender disparities in dietary intake, with males having a more Western-style diet and females a higher likelihood of following vegetarian diets [17]. Food insecurity further complicates the dietary landscape for university students in Lebanon, as it was found to be associated with a lower diet quality among university students in Lebanon [18]. that compromised food availability [2, 12]. The combined effects of Lebanon’s economic collapse, the COVID-19 pandemic, and the country’s heavy reliance on food imports, along with the devastating Beirut port explosion in 2020 have resulted in an unprecedented economic crisis [19]. The latter has severely disrupted food security in the country in terms of availability, access, and utilization.

Studies from the Middle East reveal inconsistencies in the knowledge of dietary risk factors of cancer. In Jordan, university students, particularly those in medical and science disciplines, demonstrated a solid understanding of colorectal cancer [15, 20], but this knowledge did not translate into healthier dietary behaviors. In the United Arab Emirates, university students were aware of the association between diet and cancer [21]. Students that had a low knowledge were more likely to be male, single, to have a low income, and to be pursuing an undergraduate rather than a graduate degree. In Lebanon, a cross sectional study assessing the knowledge of female university students in relevance to breast cancer prevention revealed that more that 68% had insufficient knowledge and 98% had poor dietary practices in relation to cancer prevention [22].

To our knowledge, the association between knowledge and diet behavior has not been examined thoroughly. Most studies asked isolated questions about dietary habits and knowledge rather than administering culturally-specific food questionnaires and comprehensively assessing validated diet quality indices. This gap underscores the need for more targeted research to better understand the interplay between dietary behaviors and cancer prevention knowledge in this vulnerable population. This study evaluated the knowledge of cancer prevention guidelines and their association with diet quality among a sample of university students in Beirut, Lebanon. The research team hypothesized students exhibit limited knowledge, poor diet quality, with knowledge serving as a predict diet quality.

## 2. Materials and methods

### 2.1 Study protocol

This is a cross-sectional study conducted between September 2022 and May 2023 in a private university in Beirut; students were recruited between 01 February 2023 and 28 April 2023. The study was focused in Beirut as previous research projects have highlighted the high levels of pollutants in the city and exposed significant knowledge gaps in cancer risk factors and early symptoms identification in the adult community [13, 23]. A convenience sample of students aged 17 years and above attending the Lebanese American University undergraduate, graduate, and post graduate or diploma programs in Beirut campus during the academic year of 2022/2023 was recruited. No exclusion criteria were applied.

### 2.2 Ethical considerations

The study was conducted in line with the ethical principles of the declaration of Helsinki and was approved by Lebanese American University’s Institutional Review Board (LAU.SAS.JJ1.27). Approached students were informed of the research project’s goals, benefits, and risks. Those agreeing to join the study provided a verbal informed consent.

### 2.3 Data collection

Data were collected face-to-face by five trained nutrition students in their senior year. Students were approached on campus and those agreeing to participate were interviewed in private areas. Assessments included structured questionnaire comprising sociodemographic (e.g., age, gender, education level, economic status), educational (e.g., major, years in university) and lifestyle factors (smoking, physical activity), knowledge of the association between dietary components and cancer prevention, and food intake. Students majoring in biology, nursing, nutrition or pharmacy were considered to have a health-related major. Participants’ knowledge was assessed using a questionnaire developed by the research team that focused on the relationship between dietary components and cancer prevention. Dietary factors that had a conclusive level of evidence as determined by the Continuous Update Project were included in the scoring [24]. Participants provided one of four answers on the association between dietary components and cancer (increased risk, decreased risk, had no effect on cancer, unsure). Correct and incorrect responses were awarded scores of 10 and 0 respectively, and the sum of these scores provided an overall knowledge score over 100. Participants scoring above the 60^th^ percentile for the continuous knowledge score were categorized as more knowledgeable (Kn+). The rest were classified as less knowledgeable (Kn-). The knowledge questionnaire was piloted on 10 individuals, and feedback from the pilot was incorporated to produce the final version used for data collection. Participants were asked to report on their physical activity using the International Physical Activity Questionnaire- Short Form [25, 26]. Dietary intake was assessed using the Modified Mediterranean Prime Screen (MMPS), validated among women of reproductive age in Lebanon and currently being examined in adults [27]. This diet screener, composed of 32 food items and groups, allows for the assessment of nutrient intake and diet quality. The Alternate Healthy Eating Index (AHEI) was calculated from the MMPS to assess diet quality [28]. This index provides a comprehensive measure of overall dietary quality and adherence to healthy eating guidelines and has been correlated with non-communicable diseases [29]. This validated tool evaluates the intake of fruits, vegetables, nuts, legumes, whole grains, red and processed meats, sweetened beverages, trans fat, polyunsaturated and omega-3 fatty acids, and alcohol. Omega 3 intake- included the AHEI- was not assessed in our study due to the unavailability of food composition data on it in Lebanon. The overall score of the AHEI was over 100. The questionnaire was piloted on 10 individuals; feedback from the pilot was considered to produce the final version of the questionnaire used for data collection.

### 2.4 Sample size calculation

Sample size needed for the study was calculated on Epi Info (7.2.5.0) (Epi Info™ 2021) with “good knowledge” as the outcome of interest [30]. Given the limited data available on the outcome of interest at the time of study design, sample size calculation was done based on an expected prevalence of an adequate knowledge score of 50%, a 10% margin of error, 95% confidence interval, and a design effect of 3 to account for the snowball sampling. The sample size calculated was 288 students.

### 2.5 Statistical analyses

Continuous variables were summarized using means and standard deviations and categorical variables were presented as counts and percentages. Normality distribution was visually checked using the histogram and verified by the Kolmogorov-Smirnov test. The choice of the variables to be included on the regression model was based on relevant literature, an exploratory bivariate analysis, and a sample size of ≈30 observations per variable entered into the model. For the exploratory bivariate analysis, independent-sample t-test was used to compare the mean of the AHEI between groups of gender and major, and Kruskal-Wallis Test was used to compare AHEI between groups of physical activity. Spearman correlation test was used to evaluate the association between AHEI and age, years of university studies, crowding index, and knowledge score. A multivariable linear regression analysis using the Enter method was performed, taking the AHEI as the dependent variable and variables showing a p-value less than 0.2 in the bivariate analysis as independent variables. The purposeful selection of covariates using a p value of 0.2 is in line with research by Bendel and Afifi as well as Mickey and Greenland [31, 32]. Interaction between knowledge and major was also entered into the model. Multicollinearity was assessed using Variance Inflation Factor values less than 10 indicated that the model contained no multicollinearity. Moreover, correlations were inspected and variables with a coefficient higher than 0.7 denoted high correlation; accordingly, age was removed from the model. A p-value less than 0.05 was considered significant. The data were analyzed using the Statistical Package for Social Sciences, 29.0 (IBM, Armonk, NY) (S1 File).

## 3. Results

The sample included 300 participants aged 17 to 35 (55% females, mean age: 20.4±1.2 years). The mean knowledge score was 49%±19. Kn+ participants (n = 122) had a higher level of education (university degree: 29% vs. 17%, p = 0.038), likelihood of majoring in health disciplines (22% vs. 9%, p<0.01), and a lower crowding index (0.92±0.32 vs. 0.99±0.34, p = 0.04) than their peers (**Table 1**). Kn+ participants had lower rates of smoking (29% vs. 44%, p<0.01) and physical inactivity (56% vs. 30%, p<0.01) than Kn- subjects. No differences in age, gender, and time at university between the knowledge groups were noted. The mean diet AHEI was 41%±9.4 in the overall sample and was significantly higher in the Kn+ group (40% vs. 43%, p<0.01) (**Table 1**).

**Table 1:** University students’ characteristics by knowledge level.

Variable		Total n = 300	Kn- n = 178	Kn+ n = 122	p-value
**Age (years), mean ± SD**		20.49 ± 1.28	20.5 ± 1.4	20.4 ± 1	0.342
**Gender, n (%)**	Female	166 (55)	102 (57)	64 (52)	0.407
	Male	134 (45)	76 (43)	58 (48)	
**Crowding Index, mean ± SD**		0.96 ± 0.33	0.99 ± 0.34	0.92 ± 0.32	**0.04**
**Household monthly (USD), n (%)**	<1000	87 (29)	48 (27)	39 (32)	0.442
	≥1000	123 (41)	72 (40)	51 (42)	
	Prefer not to say	90 (30)	58 (33)	32 (26)	
**Time (years) at university, mean ± SD**		2.46 ± 0.99	2.5 ± 1.1	2.4 ± 0.9	0.486
**Highest level of education achieved, n (%)**	University	66 (22)	31 (17)	35 (29)	0.038
	High school/ equivalent	234 (78)	147 (83)	87 (71)	
**Health-related major, n (%)**		43 (14)	16 (9)	27 (22)	**<0.01**
**Physical Activity, n (%)**	Low	135 (45)	99 (56)	36 (30)	**<0.01**
	Moderate	115 (38)	56 (32)	59 (48)	
	High	50 (17)	23 (13)	27 (22)	
**Smokers, n (%)**		113 (38)	78 (44)	35 (29)	**<0.01**

Abbreviations: Kn-: Less knowledgeable, Kn+: More knowledgeable

**Fig 1:**
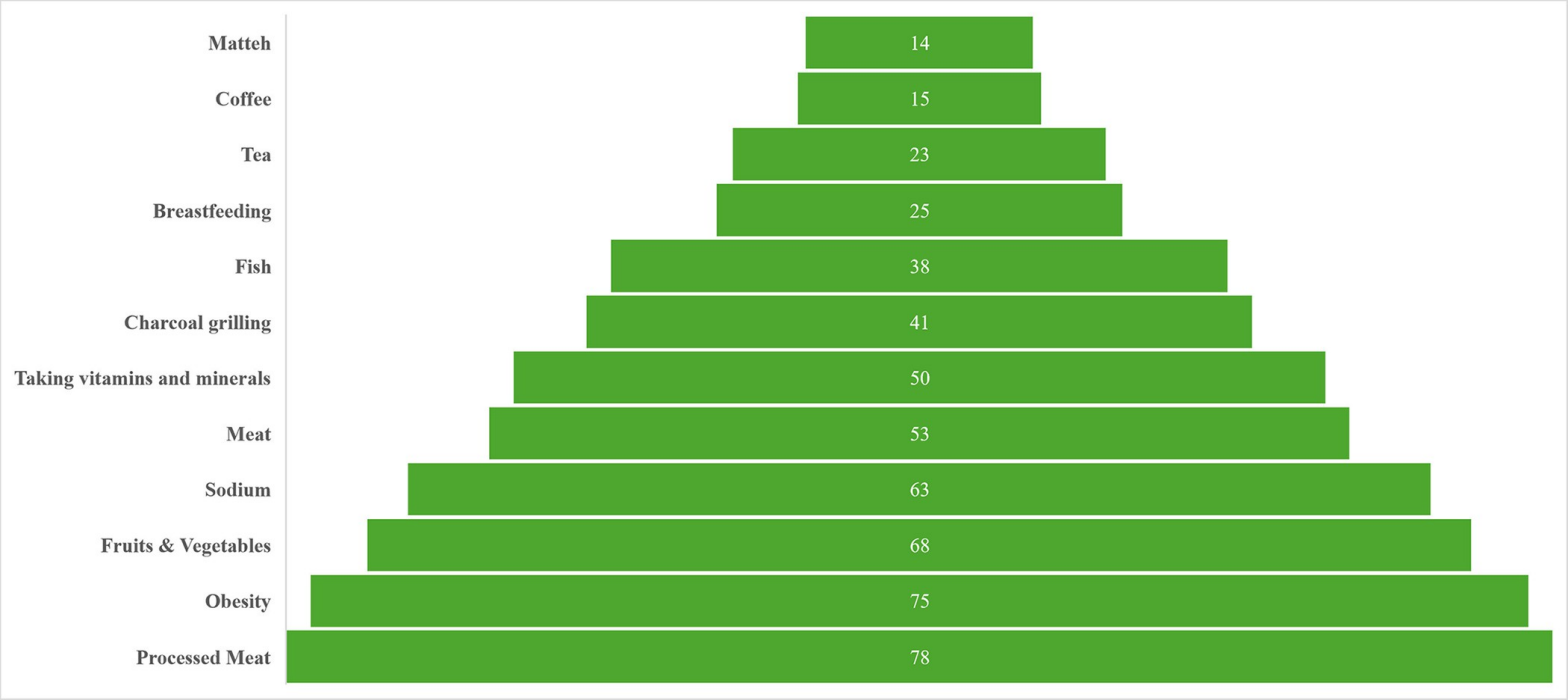
Percentage of university students knowledgeable of the correct association between dietary components and cancer.

**Fig 2:**
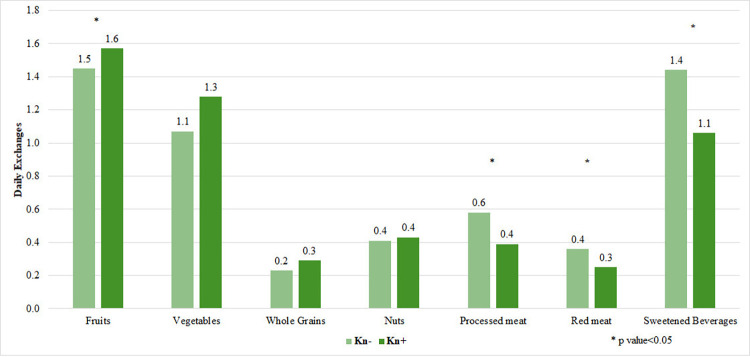
Daily exchanges of food groups consumed by knowledge categories of university students. *p value<0.05. Abbreviations: Kn-: Less knowledgeable, Kn+: More knowledgeable.

**Fig 1** features the results of the participants’ knowledge of the correct association between dietary components and cancer prevention. More than 50% of students were aware of the relationship between processed meat (78%), obesity (75%), fruits and vegetables (68%), sodium (63%), and red meat (53%) and cancer. Yet, less than 50% were knowledgeable of the association between charcoal grilling (41%), fish (38%), breastfeeding (25%), tea (23%), coffee (15%), and Matteh (14%) and cancer. Taking vitamin and mineral supplements was incorrectly thought to be a protective factor by 50% of the sample. Moreover, 75% of the sample was not aware that breastfeeding reduces cancer risk. S1 Fig highlights the answers participants provided on the knowledge questions of the association between dietary components and cancer. No consensus was found on the relationship between cancer and items such as milk, fish, tea, and pepper. **Fig 2** displays the intake of food groups by knowledge categories. Students who had an elevated knowledge score were more likely to have a higher intake of vegetables, and a lower consumption of processed and red meat, as well as sweetened beverages. No other statistically significant differences were noted between the compared groups.

The multivariable linear regression examining the predictors of the AHEI is presented in **Table 2**. A significant positive association was found between knowledge score (B = 0.78, 95%CI: 0.18,1.37), high physical activity (B = 4.62, 95%CI: 1.66,7.59) and AHEI. In contrast, a significant negative association was observed between being enrolled in a health-related major (B = -14.16, 95%CI: -23.31,-5.00) and AHEI. A significant positive interaction between knowledge and being enrolled in a health-related major (B = 2.01, 95%CI: 0.42,3.60) was identified: at low knowledge scores, the AHEI score was lower in students enrolled in a health-related major. As the knowledge score increased, the differences resolved. Overall, the regression model demonstrated limited exploratory power (adjusted R^2^ = 0.15).

**Table 2:** Multivariable linear regression of university students’ diet quality assessed using the Alternative Healthy Eating Index.

	Unstandardized Coefficients	Standardized Coefficients	95% Confidence Interval for B	
	B	Beta	Lower Bound	Upper Bound
**Gender (ref. female)**	-1.98	-0.11	-4.02	0.05
**Monthly income (ref. ≥1000$)**	-2.34	-0.12	-4.81	0.13
**Monthly income (ref. Prefer not to say)**	-1.59	-0.08	-4.28	1.10
**Time (years) at university**	-0.85	-0.09	-1.86	0.16
**Health-related major (ref. no)**	-14.16	-0.53	-23.31	-5.00
**Knowledge Score**	0.78	0.16	0.18	1.37
Knowledge* Health-related major	2.01	0.44	0.42	3.60
**Physical activity (ref. moderate)**	1.40	0.07	-0.92	3.73
**Physical activity (ref. high)**	4.62	0.18	1.66	7.59

Adjusted R^2^: 0.15

Method: Enter

## 4. Discussion

This study investigated dietary knowledge of and adherence to cancer prevention guidelines among university students in Beirut, Lebanon. Students had a limited understanding of the cancer prevention guidelines and a moderate diet quality. Participants with a higher knowledge score consumed more vegetables and fewer sweetened beverages, processed meats, and red meats. Knowledge of the cancer prevention guidelines predicted a higher diet quality, and a significant interaction was identified between having a health-related major and knowledge.

Do students have a good understanding of their dietary risk factors? Alarmingly, the mean overall knowledge score of nutritional determinants of cancer was below 50%. Half of the sample believed taking over the counter vitamins and minerals supplementation could reduce cancer incidence and 75% of participants were not aware that breastfeeding reduces cancer risk. Fortunately, more than 50% of the students were knowledgeable of the correct association between selected items such as processed meat, fruits, and cancer. This latter result is in line with findings from Saudi Arabia, Qatar, and Lebanon where more than 50% of the samples provided correct answers to similar cancer prevention questions [22, 33, 34]. In relevance to the association between knowledge and behavior, Kn+ participants consumed more vegetables and less processed meat, red meat and sweetened beverages than their peers in this research project. However, the intake of the rest of the food groups i.e., fruits, whole grains, and nuts was similar. Multivariable analysis revealed that a higher knowledge score predicted better diet quality, though the overall model was not powerful.

Studies conducted among university students in Jordan, Lebanon, Saudi Arabia, and the United Arab Emirates revealed that the knowledge of the participants did not translate into improved dietary behaviors [20–22, 34]. Similarly, it was found that having a health-related major was associated with an improved dietary knowledge rather than a superior diet quality [20, 34]. Differences in the participants’ characteristics, method of data collection such as face-to-face questionnaires vs. online electronic questionnaire, and the use of non-validated questionnaires to assess knowledge and dietary intake limited our ability to directly compare findings.

Diet is one of many modifiable risk factors of cancer incidence. An examination of the knowledge and behavior of smoking, physical activity, environmental pollutants, and timely cancer screening is needed for a comprehensive understanding of cancer determinants [7]. A study among adults in Lebanon revealed low awareness of infectious agents and work exposures as cancer risk factors [35]. Another research project assessing knowledge of cancer determinants among adults revealed that while almost all participants were aware that smoking and air pollution increase cancer risk, 49% were not aware of the harm of sedentary behavior [13]. Of interest, health literacy in this study was associated with a low-risk behavior for all factors except smoking.

Health education is essential for improving cancer literacy and diet quality. Integrating nutrition into school and university curricula positively affects dietary behavior in high school students [36]. For interventions to be effective, they need to be started at an early age and to integrate frameworks of behavioral change such as the Health Belief Model and the Transtheoretical Model, both of which have proven to be effective in promoting cancer related among adults [36, 37]. However, the determinants of healthy behavior extend beyond individual cancer literacy. Public health authorities need to deepen their understanding of the food and health systems. The World Health Organization advocates for the implementation of policies creating a healthy food environment. Examples of these reforms are the implementation of marketing restrictions on unhealthy foods and beverages for vulnerable populations, the adoption of front of pack labels to empower customers to make healthy choices, and the elimination of trans fatty acids from food products [38–40]. In Lebanon, however, these critical reforms have not been legislated and laws as basic as the adoption of back of pack labels have not been issued [40–42].

### 4.1 Strengths and limitations

This study has several strengths and limitations. The study used the MMPS, a culturally-adapted and comprehensive questionnaire to assess diet quality. Moreover, the AHEI was utilized to evaluate diet quality. This tool has been found to be highly correlated with non-communicable diseases and cancer incidence and allows for comparability with international studies [29]. The study was well powered to study the primary outcome of interest. The limitations of the study include its cross-sectional design which restricts the ability to draw a temporal association between knowledge and dietary behavior. Moreover, the researchers employed a questionnaire developed by the research team that was not validated, which may limit the reliability and generalizability of the findings. The use of convenience sampling from a single university limits the generalizability of the results to the broader Lebanese youth. Finally, the lack of anthropometric measurements limited the possibility to study the association between weight status, knowledge, and diet quality.

## 5. Conclusions

Even though cancer incidence is among the highest in the region, knowledge and behavior in relevance to cancer prevention remain limited. While greater knowledge positively influenced the consumption of certain food groups, its effect was limited. Our findings underscore the necessity for an assessment of the food systems affecting the dietary patterns of university students in Lebanon to better understand the predictors of diet quality. Future studies should employ validated and standardized tools to assess knowledge and diet quality to allow for comparability across studies. Efforts should be made to validate findings in other universities across Lebanon and the Middle East. Legal reforms are essential to empower university students to adopt healthier dietary behaviors, ultimately reducing cancer risk and promoting long-term health.

## Supporting information

S1 File Dataset. (SAV)

S1 Fig Participants’ responses on the association between selected dietary risk factors and cancer. (TIF)

## List of abbreviations

Kn: Knowledgeable
Kn: Less knowledgeable
AHEI: Alternative Healthy Eating Index
MMPS: Modified Mediterranean Prime Screen
