# Supplementary figures and images for: Suboptimal dietary knowledge predicts lower diet quality for cancer prevention among university students in Beirut
Source: PLoS One. 2025 Jan 3;20(1):e0315911. doi: 10.1371/journal.pone.0315911 (PMC11698321; doi:10.1371/journal.pone.0315911)

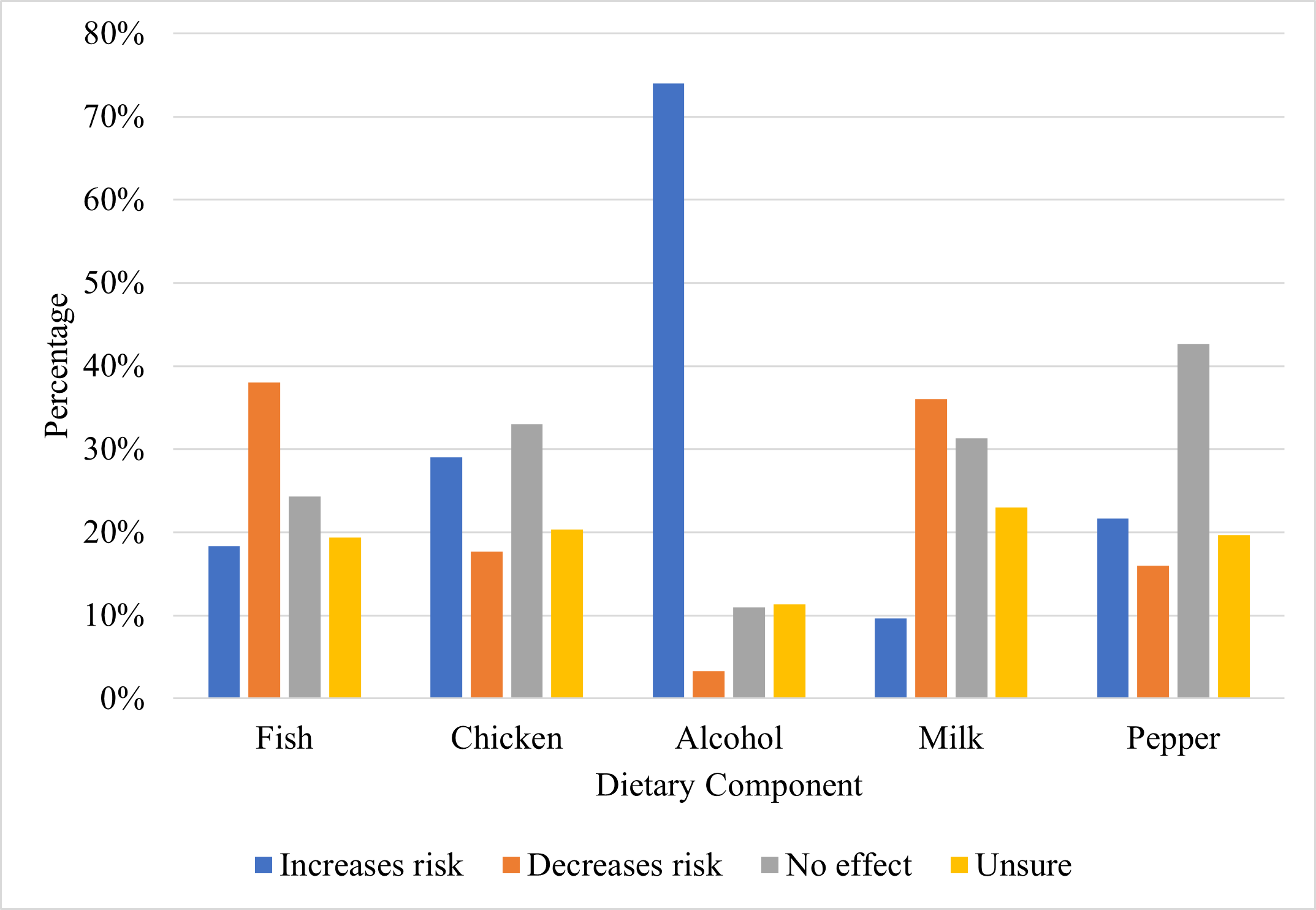

Supplement: S1 Fig — (TIF) [file pone.0315911.s002.tif]
